# Association between the Albumin-to-Globulin Ratio and Atrial Fibrillation in Patients with Hypertrophic Cardiomyopathy

**DOI:** 10.31083/j.rcm2503096

**Published:** 2024-03-07

**Authors:** Zhongyu Yuan, Ke Zhang, Haiwei Li, Shengwei Wang, Xiaoyan Li, Weiping Sun, Fei Hang, Yingchen Mei, Rui Han, Changhua Wang, Yongqiang Lai, Yongquan Wu, Xiaoping Zhang

**Affiliations:** ^1^Department of Cardiology, Beijing Anzhen Hospital, Capital Medical University, 100029 Beijing, China; ^2^Cardiovascular Surgery Center, Beijing Anzhen Hospital, Capital Medical University, 100029 Beijing, China; ^3^Beijing Anzhen Hospital, Capital Medical University, The Key Laboratory of Remodelling-Related Cardiovascular Diseases, Ministry of Education, Beijing Institute of Heart, Lung and Blood Vessel Diseases, 100029 Beijing, China; ^4^Department of Cardiology, Beijing Jishuitan Hospital, 100029 Beijing, China

**Keywords:** hypertrophic cardiomyopathy, atrial fibrillation, albumin-to-globulin ratio

## Abstract

**Background:**

Atrial fibrillation (AF), which occurs four to six times more frequently in hypertrophic cardiomyopathy (HCM) patients than in the general population, is the most common persistent arrhythmia and has a substantial therapeutic consequence. In HCM patients, there are currently no discovered signs that could be utilized to identify AF.

**Methods:**

From 2018 to 2022, 493 individuals with a continuous diagnosis of HCM were examined at Beijing Anzhen Hospital. AF was proven using routine electrocardiography (ECG), 24-hour Holter ECGs, or bedside ECGs. Echocardiography and blood tests were performed for all patients. Analysis and comparison of the traits were performed in HCM patients with AF (n = 77) and without AF (n = 416).

**Results:**

Age (*p* <0.001), prevalence of ventricular tachycardia (VT, *p* < 0.001), prevalence of pulmonary artery hypertension (*p* = 0.027), and albumin-to-globulin ratio (AGR, *p* = 0.046) were all significantly higher in patients with AF, compared to patients without AF. In multivariate logistic analysis, age (odds ratio [OR], 1.063; 95% confidence interval [CI], 1.032–1.095; *p* < 0.001), history of VT (OR, 2.702; 95% CI, 1.007–7.255; *p* = 0.048), AGR (OR, 3.477; 95% CI, 1.417–8.536; *p* = 0.007), left atrial diameter (OR, 1.132; 95% CI, 1.073–1.194; *p* < 0.001), left ventricular end-diastolic diameter (OR, 0.861; 95% CI, 0.762–0.974; *p* = 0.017), left ventricular end-systolic diameter (OR, 1.239; 95% CI, 1.083–1.417; *p* = 0.002), and peak A wave velocity (OR, 0.983; 95% CI, 0.972–0.994; *p* = 0.002) were independently associated with AF in HCM patients. In the receiver operating characteristic curve analysis, the area under the curve for the established model was 0.819 (95% CI, 0.755–0.883, *p* = 0.033), with a sensitivity and specificity of 0.763 and 0.816, respectively, for AF occurrence in HCM patients.

**Conclusions:**

In individuals with HCM, a history of VT and a higher AGR are independently linked to AF. Further investigation is necessary to determine whether increased AGR represents a risk factor for embolic stroke or cardiovascular death.

## 1. Introduction

Hypertrophic cardiomyopathy (HCM) is a prevalent hereditary cardiac disease that impacts 0.2% of the population [1]. Atrial fibrillation (AF) is undoubtedly the most prevalent persistent arrhythmia in HCM patients, with between 20% and 25% of HCM patients experiencing symptomatic episodes and a yearly rate of 2% to 4%, it represents a 4–6-fold greater prevalence than in the global population, and it has a major clinical impact [2, 3, 4, 5]. AF correlates with an increase in morbidity and death due to complications, including heart failure (HF), systemic embolism, and stroke, thereby resulting in a substantial public health burden. Once AF develops in HCM patients, the initiation of anticoagulation should be considered, irrespective of the ${\mathrm{CHA}}_{2}$${\mathrm{DS}}_{2}$-VASc score, while rhythm control for symptomatic AF and rate control for asymptomatic AF should also be prescribed [6, 7]. Early identification and effective interventions of AF in people suffering from HCM are expected to improve their prognosis and quality of life.

A larger load of atrial myopathy and fibrosis in HCM patients is believed to hinder sinus impulse propagation across the atrium, thereby providing a substrate for delayed conduction and intra-atrial re-entry [8, 9]. As the pathogenesis of AF involves inflammation, serum inflammatory markers, such as complement (C1q), albumin-to-globulin ratio (AGR), and neutrophil-to-lymphocyte ratio (NLR) may be beneficial for the identification and risk stratification of AF [10, 11, 12, 13, 14]. However, whether a link exists between the AGR and AF occurrence in HCM patients remains unclear. Therefore, to identify possible risk factors for AF and their potential interactions with the occurrence of AF in HCM patients, we analyzed the features of a cohort of HCM patients.

## 2. Methods

### 2.1 Patients

**Fig. 1.**
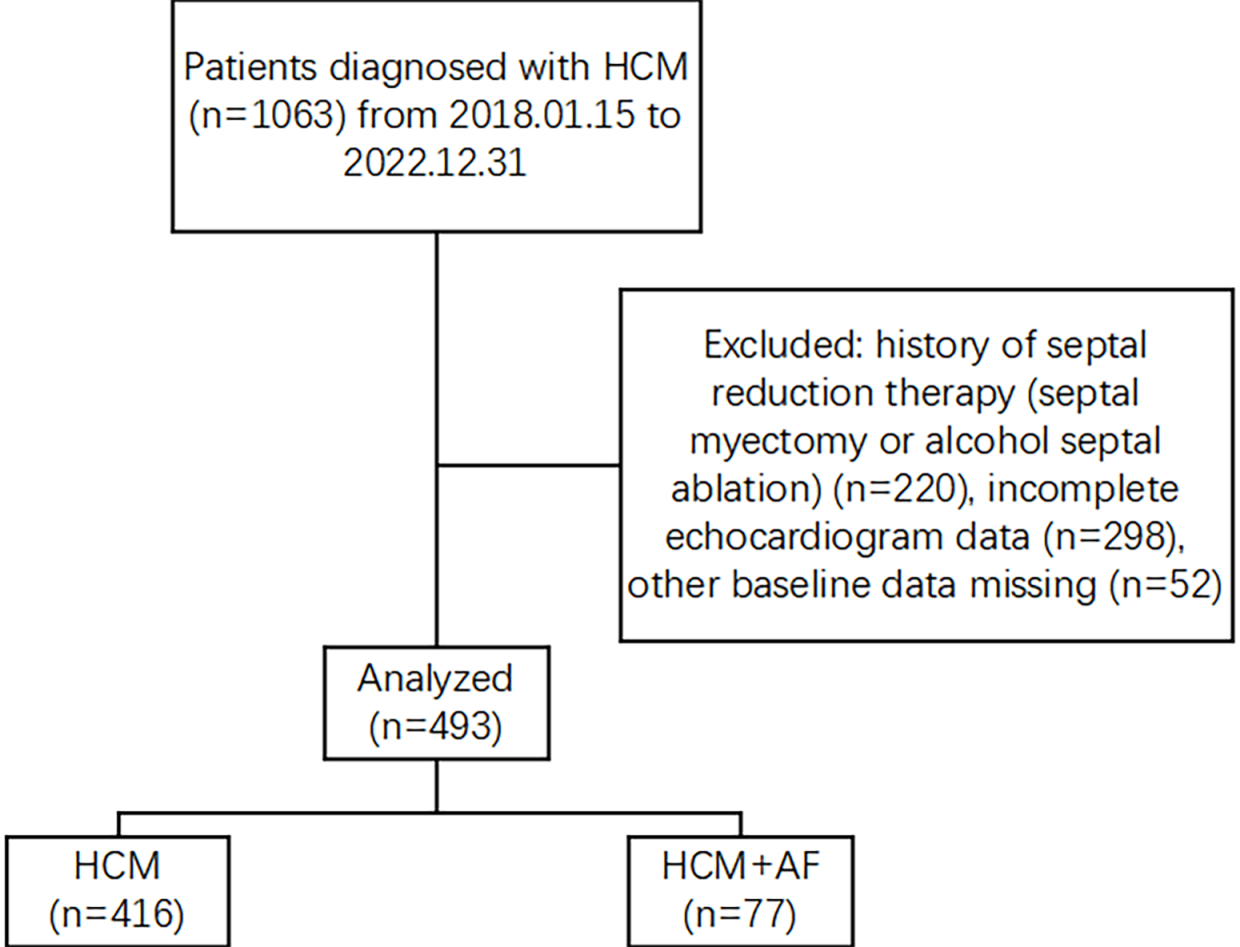
**Flowchart of the study**. HCM, hypertrophic cardiomyopathy; AF, atrial fibrillation.

Records of 1063 consecutive HCM patients at Anzhen Hospital (Beijing, China) between January 2018 and December 2022 were retrospectively evaluated for their probable inclusion in the study. Unexplained septal hypertrophy with a thickness of 15 mm was used to diagnose HCM, as indicated by the 2014 European Society of Cardiology guidelines and the 2020 American Heart Association/American College of Cardiology guidelines [6, 15]. AF was proven by routine electrocardiography (ECG), 24-hour Holter ECG, or dynamic ECG monitoring. Excluded patients were those with a history of septal reduction therapy (septal myectomy or alcohol septal ablation, n = 220), incomplete echocardiogram data (n = 298), and other baseline data missing (n = 52), meaning 493 patients comprised the final study cohort (Fig. 1).

The Ethics Committee of Beijing Anzhen Hospital (Grant No. KS2023004) authorized this prospective observational study and informed permission was provided by all patients prior to enrolling. All patient testing was conducted in conformity with the Helsinki Declaration’s ethical principles.

### 2.2 Echocardiography

One competent physician performed each echocardiographic evaluation using GE LOGIQ E9 (GE Healthcare, CA, USA) ultrasound equipment. From standardized perspectives, two-dimensional and two-dimensionally directed M-mode pictures were captured [16]. Left atrial diameter (LAD), interventricular septal thickness (IVST), left ventricular end-diastolic diameter (LVEDD), left ventricular end-systolic diameter (LVESD), left ventricular posterior wall thickness (LVPWT), left ventricular ejection fraction (LVEF), peak E wave velocity, peak A wave velocity, and E/A ratio (the ratio of early [E-wave] and late [A-wave] left ventricular diastolic filling velocities) were measured. The heart chamber diameters were determined as the highest value of the anteroposterior diameter during cardiac cycles, while IVST and LVPWT were determined during diastole. The biplane Simpson technique was used to calculate LVEF. The left ventricular mass (LVM) was estimated according to the Devereux formula: LVM (g) = 0.8 × (1.04 × (${\mathrm{(LVEDD + IVST + LVPWT)}}^{3}$ - ${\mathrm{LVEDD}}^{3}$)) + 0.6 [17]. LVM was divided by body surface area (BSA) to calculate the LVM index. The formula used to estimate BSA was as follows: 0.0073 × (height in centimeter) + 0.0127 × (weight in kilogram) – 0.2106 (for female), 0.0057 × (height in centimeter) + 0.0121 × (weight in kilogram) + 0.0882 (for male) [18]. The rate of mild to severe mitral regurgitation (MR) or pulmonary artery hypertension (PAH) was also recorded.

### 2.3 Other Clinical Indices

A standardized medical history, including New York Heart Association (NYHA) cardiac function level; an accurate physical examination, which included body mass index (BMI) and body surface area (BSA); thorough clinical interventions, including oral medications and implantable cardioverter-defibrillator (ICD) implantation, were all obtained from each patient. Routine clinical blood examinations, including mean corpuscular volume (MCV), mean corpuscular hemoglobin (MCH), red blood cell distribution width–standard deviation (RDW–SD), high-sensitivity C-reactive protein (hs-CRP), and brain natriuretic peptide (BNP); serum biochemical tests, including alanine aminotransferase (ALT), aspartate transaminase (AST), gamma-glutamyltransferase (GGT), and estimated glomerular filtration rate (eGFR), were each performed using standard assays after a 12 h fasting (no alcohol) for each patient.

### 2.4 Statistical Analysis

For continuous variables in categorical categories, the baseline characteristics were presented as mean, standard deviation, and proportions; for continuous variables that did not follow normal distributions, the median (with 25% and 75% percentiles) was presented. The unpaired Student *t*-test, Chi-square, or Fisher’s exact test were employed alongside the Mann–Whitney U test to assess any differences in normally distributed continuous, categorical, and non-normally distributed continuous data. The association between continuous factors and the incidence of AF was estimated using point-biserial correlation analysis. Chi-square tests and phi coefficient (phi) calculations were used to explore the associations between a history of ventricular tachycardia (VT) or PAH and AF occurrence. Collinearity and multicollinearity were analyzed utilizing tolerance (cutoff value <0.1) and the variance inflation factor (VIF, cutoff value >10). To identify parameters linked with AF in HCM patients, univariate and stepwise multivariate logistic regression analyses were employed. Variables with a *p* value of 0.05 in the univariate analysis were included in a multivariate analysis; all reported probability values were two-tailed, and a *p* value of 0.05 was considered statistically significant. The area under the receiver operating characteristic (ROC) curve was used to identify the cutoff value for the prediction probability based on the multivariate logistic regression model. For computations and drawings, SPSS version 28.0 (IBM Corp, Armonk, NY, USA) and Prism version 9.5.1 (GraphPad Software Inc., La Jolla, CA, USA) were used, respectively.

## 3. Results

### 3.1 Baseline Patient Characteristics

Tables 1,2,3 describe the baseline features in each subgroup, in accordance with the comorbidity of AF. Patients with AF were older (59.32 ± 10.29 versus 54.11 ± 13.75, *p* < 0.001), were more likely to experience stroke, and administered more oral medications, such as Class III antiarrhythmic drugs, anticoagulants, and antiplatelets. They had a higher prevalence of VT and were prone to ICD implantation. MCV, MCH, RDW–SD, AGR, ALT, AST, and GGT were significantly increased with AF occurrence. There were no distinctions in hs-CRP, NLR, or C1q levels between the two groups. The LAD, LVEDD, and LVESD were significantly enlarged in patients with AF, whereas LVEF and peak A wave velocity were decreased. A considerably lower E/A ratio, with a mean slightly less than 1, was found (0.97 ± 0.57 versus 1.19 ± 0.65, *p* = 0.009) in patients without AF, indicating that the impaired diastolic function was associated with left ventricular hypertrophy. Left atrial pressures rose due to reduced left ventricular filling, which was accompanied by left atrial hypertrophy and enlargement, increased atrial fibrosis, and a reduction in the intra-atrial and inter-atrial electrical conduction velocities. When it comes to an enlarged atrium, an E/A ratio greater than 1 is a pseudo-normalization, suggesting a moderate decrease in the patient’s diastolic function. Furthermore, the occurrence of AF was related to a greater incidence of PAH (19.5% versus 10.6%, *p* = 0.027). There were no significant differences between the two groups in relation to gender, BMI, systolic blood pressure, NYHA cardiac function level, BNP, uric acid, eGFR, glycosylated hemoglobin, triglyceride, total cholesterol, low-density lipoprotein cholesterol, and albumin, as well as in the clinical comorbidities, such as MR, coronary heart disease, and diabetes.

**Table 1.** **Baseline characteristics of the study population**.

	HCM (n = 416)	HCM + AF (n = 77)	*p* value
Male, n (%)	240 (57.7)	45 (58.4)	0.903
Age (y)	54.11 ± 13.75	59.32 ± 10.29	<0.001
BMI (kg/$m^{2}$)	25.82 ± 3.38	25.93 ± 3.06	0.791
BSA ($m^{2}$)	1.76 ± 0.21	1.79 ± 0.22	0.343
SBP (mmHg)	127.3 ± 18.77	125.64 ± 17.75	0.471
DBP (mmHg)	74.47 ± 11.92	72.87 ± 11.69	0.279
Hypertension, n (%)	184 (44.2)	38 (49.4)	0.407
Hyperlipidemia, n (%)	111 (26.7)	20 (26.0)	0.897
Diabetes, n (%)	65 (15.6)	13 (16.9)	0.781
Renal dysfunction	22 (5.3)	5 (6.5)	0.593
Liver disease	24 (5.8)	3 (3.9)	0.784
Coronary heart disease, n (%)	100 (24.0)	22 (28.6)	0.397
Stroke, n (%)	23 (5.5)	11 (14.3)	0.005
NYHA class II–III, n (%)	110 (26.4)	21 (27.3)	0.880
VT, n (%)	16 (3.8)	12 (15.6)	<0.001
β-blockers, n (%)	241 (57.9)	33 (42.9)	0.014
CCBs, n (%)	157 (37.7)	15 (19.5)	0.002
ACEIs/ARBs, n (%)	51 (12.3)	10 (13.0)	0.859
Diuretics, n (%)	166 (39.9)	24 (31.2)	0.148
Class III antiarrhythmic drugs, n (%)	13 (3.1)	19 (24.7)	<0.001
Anticoagulants, n (%)	95 (22.8)	30 (39.0)	0.003
Antiplatelets, n (%)	124 (29.8)	10 (13.0)	0.002
Statins (%)	122 (29.3)	19 (24.7)	0.407
ICD intervention, n (%)	21 (5.0)	9 (11.7)	0.036

The values are presented as mean SD, median (interquartile range), or n (%). HCM, hypertrophic cardiomyopathy; AF, atrial fibrillation; BMI, body mass index; BSA, body surface area; SBP, systolic blood pressure; DBP, diastolic blood pressure; NYHA, New York Heart Association; VT, ventricular tachycardia; CCBs, calcium channel blockers; ACEIs/ARBs, angiotensin-converting enzyme inhibitors/angiotensin receptor blockers; ICD, implantable cardioverter-defibrillator; SD, standard deviation.

**Table 2.** **Laboratory tests for the study population**.

Laboratory test	HCM (n = 416)	HCM + AF (n = 77)	*p* value
Fasting plasma glucose (mmol/L)	5.85 ± 2.16	5.77 ± 1.7	0.767
Glycosylated hemoglobin (%)	6.19 ± 1.3	6.11 ± 0.75	0.621
Triglyceride (mmol/L)	1.65 ± 1	1.83 ± 1.41	0.221
Total cholesterol (mmol/L)	4.44 ± 0.97	4.32 ± 1.04	0.374
HDL-C (mmol/L)	1.09 ± 0.25	1.06 ± 0.24	0.477
LDL-C (mmol/L)	2.71 ± 0.81	2.56 ± 0.94	0.213
SdLDL (mmol/L)	0.76 ± 0.36	0.73 ± 0.34	0.561
Non-HDL-C (mmol/L)	3.37 ± 0.96	3.24 ± 0.98	0.468
FFA (mmol/L)	0.41 ± 0.22	0.44 ± 0.19	0.354
Complement [C1q] (mg/L)	174.04 ± 36.36	166.57 ± 40.03	0.231
Urea nitrogen (mmol/L)	6.56 ± 2.62	5.98 ± 2.73	0.111
Uric acid (µmol/L)	377.76 ± 97.78	361.19 ± 82.71	0.218
Creatinine (mmol/L)	81.7 ± 74.31	78.74 ± 38.15	0.759
eGFR (mL/min per 1.73 $m^{2}$)	92.03 ± 21.9	87.38 ± 18.17	0.115
BNP (pg/mL)	379.5 (174.25, 845.75)	438 (207, 606)	0.767
Hs-CRP (mg/L)	0.90 (0.5, 2.04)	1.03 (0.51, 1.73)	0.945
Neutrophil (${\mathrm{10}}^{9}$/L)	5.33 ± 3.36	4.81 ± 2.26	0.123
Lymphocyte (${\mathrm{10}}^{9}$/L)	1.79 ± 0.76	2.20 ± 2.01	0.125
NLR	2.25 (1.56, 3.23)	1.99 (1.46, 3.97)	0.476
Red blood cell (${\mathrm{10}}^{12}$/L)	4.37 ± 0.75	4.42 ± 0.64	0.620
Hemoglobin (g/dL)	132.92 ± 24.89	136.45 ± 22.7	0.295
MCV (fL)	88.22 ± 5.59	89.93 ± 5.5	0.026
MCH (pg)	30.39 ± 2.41	31.07 ± 2.56	0.040
MCHC (g/L)	344.35 ± 14.27	345.13 ± 15.04	0.692
RDW–SD (fL)	42.43 ± 4.74	43.76 ± 3.57	0.035
Platelet (${\mathrm{10}}^{9}$/L)	188.46 ± 66.68	188.6 ± 68.61	0.988
ALT (U/L)	18 (13, 26)	23 (15, 30)	0.034
AST (U/L)	20 (17, 24)	22 (18, 26)	0.040
Albumin (g/L)	42.47 ± 3.82	42.49 ± 4.29	0.968
AGR	1.72 ± 0.32	1.81 ± 0.35	0.046
Total bilirubin (µmol/L)	13.43 ± 6.02	15.66 ± 9.21	0.077
Direct bilirubin (µmol/L)	4.10 ± 2.11	5.17 ± 4.5	0.078
Alkaline phosphatase (U/L)	74.50 ± 32.35	69.34 ± 21.85	0.237
GGT (U/L)	24 (17, 36)	27 (21, 40)	0.018
Total bile acid (µmol/L)	3.2 (2.1, 5.58)	3.5 (2.4, 7.1)	0.140
Cholinesterase (kU/L)	7.93 ± 1.8	7.55 ± 2.03	0.138

The values are presented as mean SD, median (interquartile range), or n (%). HCM, hypertrophic cardiomyopathy; AF, atrial fibrillation; HDL-C, high-density lipoprotein cholesterol; LDL-C, low-density lipoprotein cholesterol; SdLDL, small dense LDL; Non-HDL-C, non-high-density lipoprotein cholesterol, was calculated by subtracting HDL-C from total cholesterol; FFA, free fatty acid; eGFR, estimated glomerular filtration rate; BNP, brain natriuretic peptide; Hs-CRP, high-sensitivity C-reactive protein; NLR, neutrophil-to-lymphocyte ratio; MCV, mean corpuscular volume; MCH, mean corpuscular hemoglobin; MCHC, mean corpuscular hemoglobin concentration; RDW–SD, red blood cell distribution width–standard deviation; SD, standard deviation; ALT, alanine aminotransferase; AST, aspartate transaminase; AGR, albumin-to-globulin ratio; GGT, gamma-glutamyl transferase.

**Table 3.** **Echocardiographic indices in the study population**.

Echocardiographic indices	HCM (n = 416)	HCM + AF (n = 77)	*p* value
MR, n (%)	357 (85.8)	71 (92.2)	0.128
PAH, n (%)	44 (10.6)	15 (19.5)	0.027
LAD (mm)	41.56 ± 5.82	45.71 ± 6.48	<0.001
IVST (mm)	19.84 ± 4.87	18.74 ± 3.85	0.060
LVEDD (mm)	43.21 ± 5.47	45 ± 5.9	0.010
LVESD (mm)	26.94 ± 4.79	29.38 ± 5.26	<0.001
LVPWT (mm)	12.44 ± 3.24	11.79 ± 2.75	0.068
LVM (g)	298.45 ± 102.41	285.87 ± 73.78	0.201
LVM index (g/$m^{2}$)	170 ± 56.94	160.87 ± 40.39	0.092
LVEF (%)	66.25 ± 6.95	62.58 ± 8.39	<0.001
Peak E wave velocity (cm/s)	80.48 ± 30.16	78.35 ± 36.41	0.583
Peak A wave velocity (cm/s)	92 ± 30.42	75.97 ± 32.99	<0.001
E/A ratio	0.97 ± 0.57	1.19 ± 0.65	0.009

The values are presented as mean SD, median (interquartile range), or n (%). HCM, hypertrophic cardiomyopathy; AF, atrial fibrillation; MR, mitral regurgitation; PAH, pulmonary artery hypertension; LAD, left atrial diameter; IVST, interventricular septal thickness; LVEDD, left ventricular end-diastolic diameter; LVESD, left ventricular end-systolic diameter; LVPWT, left ventricular posterior wall thickness; LVM, left ventricular mass; LVEF, left ventricular ejection fraction; SD, standard deviation.

### 3.2 Clinical Data Associated with AF

Thereafter, we explored the relationship between the aforementioned clinical parameters and statistically significant intergroup differences in AF occurrence in HCM patients. The correlation coefficients were all less than 0.3, which is suggestive of a weak correlation (Table 4).

**Table 4.** **Correlation between clinical parameters and AF using point-biserial coefficients and phi coefficients**.

	Point-biserial	*p* value
Age	0.142	0.002
MCV	0.105	0.026
MCH	0.098	0.040
RDW–SD	0.100	0.035
AGR	0.096	0.046
ALT	–0.010	0.838
AST	–0.006	0.903
GGT	0.110	0.022
LAD	0.247	<0.001
LVEDD	0.117	0.010
LVESD	0.180	<0.001
LVEF	–0.182	<0.001
Peak A wave velocity	–0.186	<0.001
E/A ratio	0.131	0.003
	Phi	
VT	0.184	<0.001
PAH	0.100	0.027

AF, atrial fibrillation; MCV, mean corpuscular volume; MCH, mean corpuscular hemoglobin; RDW–SD, red blood cell distribution width–standard deviation; AGR, albumin-to-globulin ratio; ALT, alanine aminotransferase; AST, aspartate transaminase; GGT, gamma-glutamyl transferase; LAD, left atrial diameter; LVEDD, left ventricular end-diastolic diameter; LVESD, left ventricular end-systolic diameter; LVEF, left ventricular ejection fraction; VT, ventricular tachycardia; PAH, pulmonary artery hypertension.

The univariate and multivariate logistic regression analysis findings on HCM patients with and without AF are shown in Table 5. The results of the univariate logistic regression analysis indicated age (odds ratio [OR], 1.033; 95% confidence interval [CI], 1.012–1.054; *p* = 0.002), LAD (OR, 1.113; 95% CI, 1.069–1.158; *p* < 0.001), LVEDD (OR, 1.061; 95% CI, 1.014–1.109; *p* = 0.01), LVESD (OR, 1.103; 95% CI, 1.05–1.158; *p* < 0.001), LVEF (OR, 0.937; 95% CI, 0.908–0.968; *p* < 0.001), peak A wave velocity (OR, 0.981; 95% CI, 0.973–0.99; *p* < 0.001), E/A ratio (OR, 1.66; 95% CI, 1.158–2.381; *p* = 0.006), MCV (OR, 1.068; 95% CI, 1.008–1.131; *p* = 0.025), MCH (OR, 1.161; 95% CI, 1.008–1.338; *p* = 0.039), AGR (OR, 2.223; 95% CI, 1.006–4.909; *p* = 0.048), VT (OR, 4.615; 95% CI, 2.088–10.201; *p* < 0.001), and PAH (OR, 2.045; 95% CI, 1.073–3.898; *p* = 0.03) were strongly linked to the development of AF.

**Table 5.** **Univariate logistic regression analysis of significant variables linked to AF occurrence**.

	*p* value	OR	95% CI
Univariate			
Age	0.002	1.033	1.012–1.054
LAD	<0.001	1.113	1.069–1.158
LVEDD	0.010	1.061	1.014–1.109
LVESD	<0.001	1.103	1.050–1.158
LVEF	<0.001	0.937	0.908–0.968
Peak A wave velocity	<0.001	0.981	0.973–0.990
E/A ratio	0.006	1.660	1.158–2.381
MCV	0.025	1.068	1.008–1.131
MCH	0.039	1.161	1.008–1.338
RDW–SD	0.059	1.050	0.998–1.103
AGR	0.048	2.223	1.006–4.909
GGT	0.046	1.005	1.000–1.011
VT	<0.001	4.615	2.088–10.201
PAH	0.030	2.045	1.073–3.898

AF, atrial fibrillation; LAD, left atrial diameter; LVEDD, left ventricular end-diastolic diameter; LVESD, left ventricular end-systolic diameter; LVEF, left ventricular ejection fraction; MCV, mean corpuscular volume; MCH, mean corpuscular hemoglobin; RDW–SD, red blood cell distribution width–standard deviation; AGR, albumin-to-globulin ratio; GGT, gamma-glutamyl transferase; VT, ventricular tachycardia; PAH, pulmonary artery hypertension; OR, odds ratio; CI, confidence interval.

**Supplementary Table 1** displays the results of collinearity between variables in the regression model. In the multivariate logistic regression model, we found that age (OR, 1.06; 95% CI, 1.026–1.095; *p* < 0.001), history of VT (OR, 4.156; 95% CI, 1.431–12.066; *p* = 0.009), AGR (OR, 2.867; 95% CI, 1.116–7.366; *p* = 0.029), LAD (OR, 1.122; 95% CI, 1.058–1.189; *p* < 0.001), LVEDD (OR, 0.84; 95% CI, 0.717–0.984; *p* = 0.031), LVESD (OR, 1.261; 95% CI, 1.026–1.551; *p* = 0.028), and peak A wave velocity (OR, 0.982; 95% CI, 0.969–0.996; *p* = 0.011) were independently associated with the onset of AF, after adjusting for MCV, MCH, PAH, E/A ratio, and LVEF, which were associated with AF in the univariable analysis. The original model removed all variables with unadjusted associations and *p* values greater than or equal to 0.05, as shown in Table 6.

**Table 6.** **Multiple logistic regression between AF occurrence and significant variables using correlation analysis**.

	*p* value	OR	95% CI
Multivariate			
Model 1			
VT	0.009	4.156	1.431–12.066
AGR	0.029	2.867	1.116–7.366
Age	<0.001	1.060	1.026–1.095
PAH	0.517	1.337	0.555–3.222
LAD	<0.001	1.122	1.058–1.189
LVEDD	0.031	0.840	0.717–0.984
LVESD	0.028	1.261	1.026–1.551
LVEF	0.947	0.998	0.930–1.070
Peak A wave velocity	0.011	0.982	0.969–0.996
E/A ratio	0.969	1.011	0.578–1.771
MCV	0.883	0.991	0.875–1.121
MCH	0.467	1.107	0.842–1.455
Model 2			
VT	0.048	2.702	1.007–7.255
AGR	0.007	3.477	1.417–8.536
Age	<0.001	1.063	1.032–1.095
LAD	<0.001	1.132	1.073–1.194
LVEDD	0.017	0.861	0.762–0.974
LVESD	0.002	1.239	1.083–1.417
Peak A wave velocity	0.002	0.983	0.972–0.994

AF, atrial fibrillation; VT, ventricular tachycardia; AGR, albumin-to-globulin ratio; PAH, pulmonary artery hypertension; LAD, left atrial diameter; LVEDD, left ventricular end-diastolic diameter; LVESD, left ventricular end-systolic diameter; LVEF, left ventricular ejection fraction; MCV, mean corpuscular volume; MCH, mean corpuscular hemoglobin; OR, odds ratio; CI, confidence interval.

**Fig. 2.**
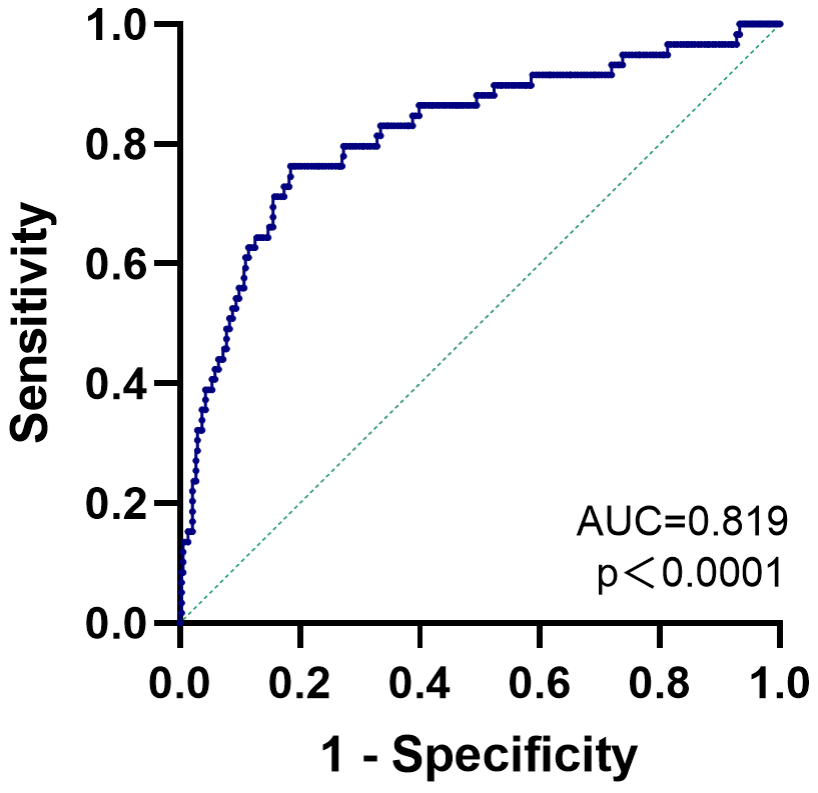
**The receiver operating characteristic (ROC) curve of the multivariate logistic regression model for predicting AF**. AF, atrial fibrillation; AUC, area under receiver operator characteristic curve.

ROC curve analysis was conducted to examine the capacity of the established multivariate logistic regression model (Model 2) to identify the presence of AF in HCM patients, and the results are displayed in Fig. 2. The area under the curve was 0.819 (95% confidence interval: 0.755–0.883, *p* = 0.033). A prediction probability of 0.158 was shown as the best cutoff point for predicting AF in HCM patients, with a sensitivity of 0.763 and a specificity of 0.816.

## 4. Discussion

### 4.1 Clinical Implications

This study conveys several new findings. First, the in-hospital incidence of AF in our research population was 15.6%, which is less than previously reported. For example, in a previous meta-analysis of 21,887 individuals from 36 cohorts with an average follow-up of 6.9 years, the presumed pooled frequency of AF among HCM patients was 22.3%, and the mean prevalence of AF was 2.5 cases per person/year [19]. One possible reason for this discrepancy is that dynamic electrocardiographic monitoring was not performed on all patients and only inpatients were enrolled in our study. Hence, the use of prolonged Holter electrocardiogram monitoring would have been more beneficial in detecting asymptomatic AF, while the simultaneous enrollment of outpatients would also avoid patient selection bias, to a certain extent. Since the risk of disability or fatal thromboembolic stroke is increased in HCM patients developing AF, along with functional decline from advancing HF, accurate risk stratification for AF is necessary to facilitate effective treatment [4, 20].

Second, we found that a history of VT and an increased level of AGR were related to a higher occurrence of AF. Although several risk models, such as the CHARGE-AF, ${\mathrm{CHA}}_{2}$${\mathrm{DS}}_{2}$-VASc, and C2HEST scores, have been constructed for the risk stratification of AF occurrence in general cardiovascular populations, their predictive accuracy in HCM patients is substantially lower [21, 22, 23]. Similarly, a left atrium (LA) transverse dimension ≥45 mm, which was recommended as a predictor of AF development in HCM in the 2014 European Society of Cardiology HCM management guidelines, has shown low sensitivity and poor discrimination in an external validation cohort [15]. Left atrial volume indexed to BSA ≥37 mL/$m^{2}$ has been an independent and more robust predictor of AF than LAD, with a negative predictive value of 92% [24]. The HCM-AF score, which combines LAD, NYHA functional class, age at HCM diagnosis, and clinical assessment, was recently developed and exceeded the previous measures in consistently classifying those with HCM for the likelihood of newly identified AF [25]. Moreover, as AF is frequently the earliest manifestation of hereditary HCM in patients without a cardiomyopathy phenotype, genetic testing may be beneficial to detect early-onset AF in this population [26]. Combining the histories of VT and AGR may allow for the prompt identification and management of this possibly fatal complication.

### 4.2 Potential Mechanisms

In multivariate logistic analysis, age, history of VT, AGR, LAD, LVEDD, LVESD, and peak A wave velocity were independently linked to AF in HCM patients. Age, as is widely known, is a demographic risk indicator for AF. HCM is distinguished by myocyte hypertrophy, myocyte disorganization, and interstitial fibrosis, resulting in a thickened wall and narrowed lumen with diastolic dysfunction [27]. The majority of individuals had HCM before developing AF, thereby demonstrating that the structural and physiological alterations are linked to the onset of AF [28]. Aside from intrinsic atrial myopathy, HCM patients are hypothesized to be predisposed to AF through an increase in the left atrial dilatation caused by left ventricular diastolic failure and MR (frequently coupled with the systolic anterior motion of the valve) [8]. Collagen cross-linking was shown to be strongly expressed in AF patients and was also connected to left atrial remodeling [29]. While a preserved E/A ratio may be due to pseudo-normalization, there were other evident signs of diastolic dysfunction, with decreases in the peak velocity of the late filling wave (A wave), which is indicative of an impairment in the atrial systolic contraction. The AF group had a slightly inferior cardiac systolic performance than the non-AF group, as evidenced by its larger LVEDD and LVESD, thereby suggesting that ventricular remodeling and altered mechanics may also predispose patients with HCM to AF. A retrospective study has proven that the rising prevalence of AF was in accordance with LV geometric remodeling patterns involving a larger LA size and a lower LVEF [30].

Myocyte hypertrophy and disarray and fibrosis serving as electrophysiological substrates may occur simultaneously within both atria and ventricles; thus, it would be reasonable to hypothesize that a history of VT is a potential indicator for AF development. The maximal diastolic potential is lower than usual in the high-pressure dilated left atrium, and myocytes are more quickly depolarized, which enhances the heart’s vulnerability to AF [31]. Fibrosis disrupts myocyte electrical connection, resulting in delayed intra- and interatrial conduction times and uneven sinus impulse propagation [32]. Ventricular fibrosis was reported to be increased in canine models of chronic AF, caused by fast atrial pacing, while AF with a quick ventricular response further boosts atrial and ventricular fibrosis [33]. In a retrospective research study, AF was linked to a higher incidence of recurrent VTs in secondary-preventive ICD participants [34]. The underlying pathophysiological mechanism of the interaction between AF and VT involved an ion channel mutation, diffuse fibrosis, sympathoexcitation, and proarrhythmic short–long–short sequences [35].

A number of inflammatory biomarkers were found to be intimately linked to AF. Plasma C1q concentrations in AF patients were considerably lower than in the controls, and in persistent AF, were lower than in paroxysmal AF [10]. NLR may be regarded as a supplemental risk evaluation tool, particularly for AF patients with a ${\mathrm{CHA}}_{2}$${\mathrm{DS}}_{2}$-VASc score below two, because the likelihood of a stroke in AF patients with NLR ≥3 was 1.4 times greater than in those with an NLR <3 [12]. The present study found no significant difference in C1q or NLR between the two groups owing to an upregulation in the systemic inflammation in HCM patients. It has been well described for human cancers that low pretreatment AGR is linked with poor overall survival (OS), disease-free survival, and progression-free survival, alongside an increased 5-year mortality [36]. Lower AGR was tightly associated with 90-day and 1-year mortalities in HF patients with decreased LVEF, indicating a role for AGR in cardiovascular protection [37]. A multivariate Cox regression analysis revealed that increased AGR was substantially linked with better OS in HF patients [38]. However, the variation tendency in AGR in patients with HCM and AF remains unclear. AGR was an independent risk factor associated with AF recurrence after cryoballoon ablation and a predictor of cardioembolic stroke [13, 14]. A prospective cohort research study also found that low albumin levels were substantially related to the occurrence of new-onset AF, as well as a 14% decrease in hazard for each extra 1 g/L of albumin [39]. Nonetheless, in the current investigation, AGR was significantly higher in the AF group than in the non-AF group, whereas there was no difference in albumin between the two groups owing mostly to the low blood globulin levels in the AF group. Globulin is composed of various proteins involved in inflammation, including complements, interleukin-6 (IL-6), and immunoglobulins, and is also known as the main executor of immune function [40]. Immune-inflammatory injury can result in detrimental remodeling. Kuusisto *et al*. [41] discovered proinflammatory immune cells infiltrating the myocardium of HCM patients, with the extent of infiltration found to be closely related to the degree of cardiac fibrosis, thereby implying that immune-inflammatory damage is a critical process that leads to the development of cardiac fibrosis in HCM. Similarly, Fang *et al*. [42] discovered that individuals with HCM had greater concentrations of proinflammatory cytokines, such as tumor necrosis factor and IL-6, in their peripheral blood serum, compared with healthy people. Increased matrix deposition after the recruitment of leukocytes and the formation of reactive oxygen species, cytokines, and growth factors result in unfavorable atrial remodeling, implying that inflammatory pathways are a precursor for AF [43]. However, there is no direct evidence to explain why blood globulin levels were relatively low in the AF group and more investigation is required to determine the role AGR plays as an indicator in the prediction of AF development.

### 4.3 Study Limitations

There are several limitations to the current investigation. First, the study was cross-sectional and single-center, meaning we cannot confirm that the increase in AGR is a risk factor for AF in HCM. Second, not all patients underwent 24-hour Holter ECG or dynamic ECG monitoring, meaning the prevalence of asymptomatic AF might have been underestimated. Third, it was impossible to ensure that every single confounding factor was adequately controlled for in the multivariate analysis. Lastly, the selected group of inpatients is likely to have more severe symptoms, which may result in bias.

## 5. Conclusions

In HCM patients, both a history of VT and a higher AGR were independently related to an increased chance and incidence of AF. The current study is the first to reveal a link between AGR and the incidence of AF in HCM patients. However, the long-term clinical association and prognostic value of a history of VT and higher AGR in AF is unknown and warrants further investigations.

## Data Availability

The datasets used and analyzed during the current study are available from the corresponding author YW on reasonable request.

## Supplementary Material

Supplementary material associated with this article can be found, in the online version, at https://doi.org/10.31083/j.rcm2503096.
