# Supplementary material for: Association between the Albumin-to-Globulin Ratio and Atrial Fibrillation in Patients with Hypertrophic Cardiomyopathy
Source: Rev Cardiovasc Med. 2024 Mar 7;25(3):96. doi: 10.31083/j.rcm2503096 (PMC11263833; doi:10.31083/j.rcm2503096)
Supplement: Supplementary file 1 [file 2153-8174-25-3-096-s1.docx]

Supplementary Table 1. Collinearity detection between variables included in the regression model.

|  | Tolerance | VIF |
| --- | --- | --- |
| Model 1 |  |  |
| Age | 0.758 | 1.32 |
| VT | 0.949 | 1.053 |
| PAH | 0.88 | 1.136 |
| LAD | 0.807 | 1.239 |
| LVEDD | 0.176 | 5.678 |
| LVESD | 0.132 | 7.554 |
| LVEF | 0.46 | 2.172 |
| Peak A wave velocity | 0.591 | 1.691 |
| E/A ratio | 0.631 | 1.585 |
| MCV | 0.215 | 4.643 |
| MCH | 0.217 | 4.618 |
| AGR | 0.899 | 1.113 |
| Model 2 |  |  |
| Age | 0.824 | 1.213 |
| VT | 0.966 | 1.035 |
| LAD | 0.893 | 1.12 |
| LVEDD | 0.253 | 3.952 |
| LVESD | 0.266 | 3.753 |
| Peak A wave velocity | 0.817 | 1.225 |
| AGR | 0.956 | 1.046 |

AGR, albumin-to-globulin ratio; MCH, mean corpuscular hemoglobin; MCV, mean corpuscular volume; LAD, left atrial diameter; LVEDD, left ventricular end-diastolic diameter; LVEF, left ventricular ejection fraction; LVESD, left ventricular end-systolic diameter; PAH, pulmonary artery hypertension; VIF, variance inflation factor; VT, ventricular tachycardia.
